# A Molecular Mechanism for Eflornithine Resistance in African Trypanosomes

**DOI:** 10.1371/journal.ppat.1001204

**Published:** 2010-11-24

**Authors:** Isabel M. Vincent, Darren Creek, David G. Watson, Mohammed A. Kamleh, Debra J. Woods, Pui Ee Wong, Richard J. S. Burchmore, Michael P. Barrett

**Affiliations:** 1 Faculty of Biomedical and Life Science and Wellcome Trust Centre for Molecular Parasitology, Glasgow Biomedical Research Centre, University of Glasgow, Glasgow, United Kingdom; 2 Strathclyde Institute for Pharmacy and Biomedical Sciences, University of Strathclyde, Glasgow, United Kingdom; 3 Pfizer Animal Health, Pfizer Inc, Kalamazoo, Michigan, United States of America; Seattle Biomedical Research Institute, United States of America

## Abstract

Human African trypanosomiasis, endemic to sub-Saharan Africa, is invariably fatal if untreated. Its causative agent is the protozoan parasite *Trypanosoma brucei*. Eflornithine is used as a first line treatment for human African trypanosomiasis, but there is a risk that resistance could thwart its use, even when used in combination therapy with nifurtimox. Eflornithine resistant trypanosomes were selected *in vitro* and subjected to biochemical and genetic analysis. The resistance phenotype was verified *in vivo*. Here we report the molecular basis of resistance. While the drug's target, ornithine decarboxylase, was unaltered in resistant cells and changes to levels of metabolites in the targeted polyamine pathway were not apparent, the accumulation of eflornithine was shown to be diminished in resistant lines. An amino acid transporter gene, *TbAAT6* (Tb927.8.5450), was found to be deleted in two lines independently selected for resistance. Ablating expression of this gene in wildtype cells using RNA interference led to acquisition of resistance while expression of an ectopic copy of the gene introduced into the resistant deletion lines restored sensitivity, confirming the role of TbAAT6 in eflornithine action. Eflornithine resistance is easy to select through loss of a putative amino acid transporter, *TbAAT6*. The loss of this transporter will be easily identified in the field using a simple PCR test, enabling more appropriate chemotherapy to be administered.

## Author Summary

We have found that the loss of a single gene, *TbAAT6*, is sufficient to render African trypanosomes resistant to the only safe drug, eflornithine, in use against them. The fact that parasites lacking *TbAAT6* are viable in animals and retain the resistance phenotype indicates a simple means by which parasite populations could develop resistance. The loss of this gene can be detected by PCR apparatus, offering the potential for a simple, cheap test in the field, meaning that the drug will not be prescribed when it would be inefficient. It will be critical to monitor parasite populations in endemic regions for the status of this gene as eflornithine is used increasingly in trypanosomiasis therapy.

## Introduction

Human African trypanosomiasis (HAT) is a neglected tropical infectious disease transmitted by biting tsetse flies and is prevalent in sub-Saharan Africa [1], [2]. In humans, the disease is caused by two sub-species of the protozoan *Trypanosoma brucei* – *T. b. gambiense* and *T. b. rhodesiense*. *T. b. gambiense* is responsible for around 95% of all cases of the disease. An alarming resurgence of the disease in the latter part of the twentieth century stimulated a renewed interest in HAT control [2].

There are two stages of HAT. The first stage is characterised by parasite proliferation in the blood and lymph, while the second stage occurs when parasites enter the CSF (cerebrospinal fluid) and brain, resulting in symptoms that include confusion, depression, personality changes and the altered sleep-wake patterns that give the disease its common name of sleeping sickness. Death follows, inevitably, without treatment. Chemotherapy in stage two HAT requires melarsoprol, a melaminophenyl arsenical, or eflornithine, an amino acid analogue which inhibits the polyamine biosynthetic enzyme ornithine decarboxylase (ODC).

Melarsoprol is exceedingly toxic, killing 5% of recipient HAT patients [2]. Furthermore, treatment failure with melarsoprol has led to its being superseded by eflornithine. Recently, nifurtimox use with eflornithine has been recommended [3], [4] and the combination added to the WHO list of essential medicines.

Eflornithine targets ornithine decarboxylase in trypanosomes [5], [6], and this causes diminished polyamine biosynthesis [5] and reduced production of the trypanosome specific redox active metabolite trypanothione [7]. Accumulation of S-adenosyl methionine has been reported in eflornithine treated cells, which might perturb cellular methylation reactions [8] although recent data identified increased levels of decarboxylated S-adenosyl methionine, but not its precursor [9]. How eflornithine enters trypanosomes is a subject of debate. An early report that eflornithine uptake by trypanosomes was not saturable established the idea that eflornithine enters trypanosomes by passive diffusion [10]. However, studies on eflornithine resistant procyclic trypanosomes showed reduced accumulation of eflornithine [11] and uptake of eflornithine was by a saturable process typical of a transporter. Bellofatto *et al* [12] also found uptake of eflornithine to be temperature dependent and thus likely to be transporter mediated. Indeed as a zwitterionic, charged amino acid, eflornithine would not be expected to diffuse across membranes and transport mediated uptake would be a pre-requisite for uptake. In *T. brucei* loss of transport has been shown to be a key determinant in resistance to melaminophenylarsenicals [13] and diamidine drugs [14]–[16].

Given the increased use of eflornithine, alone or in combination with nifurtimox, a better understanding of the risk of resistance is critical. Such an understanding may help limit its spread and allow the development of diagnostic tools such as those described for melarsoprol resistance [15], [16].

We have investigated the mechanism of resistance to eflornithine and show that acquisition of selected resistance is accompanied by loss of a specific transporter. We further show, using genetic manipulation, that this transporter mediates uptake of eflornithine and that its loss confers resistance, whilst its expression in resistant lines restores sensitivity.

## Results

### Selection of eflornithine resistant bloodstream form *T. brucei*

**Figure 1:**
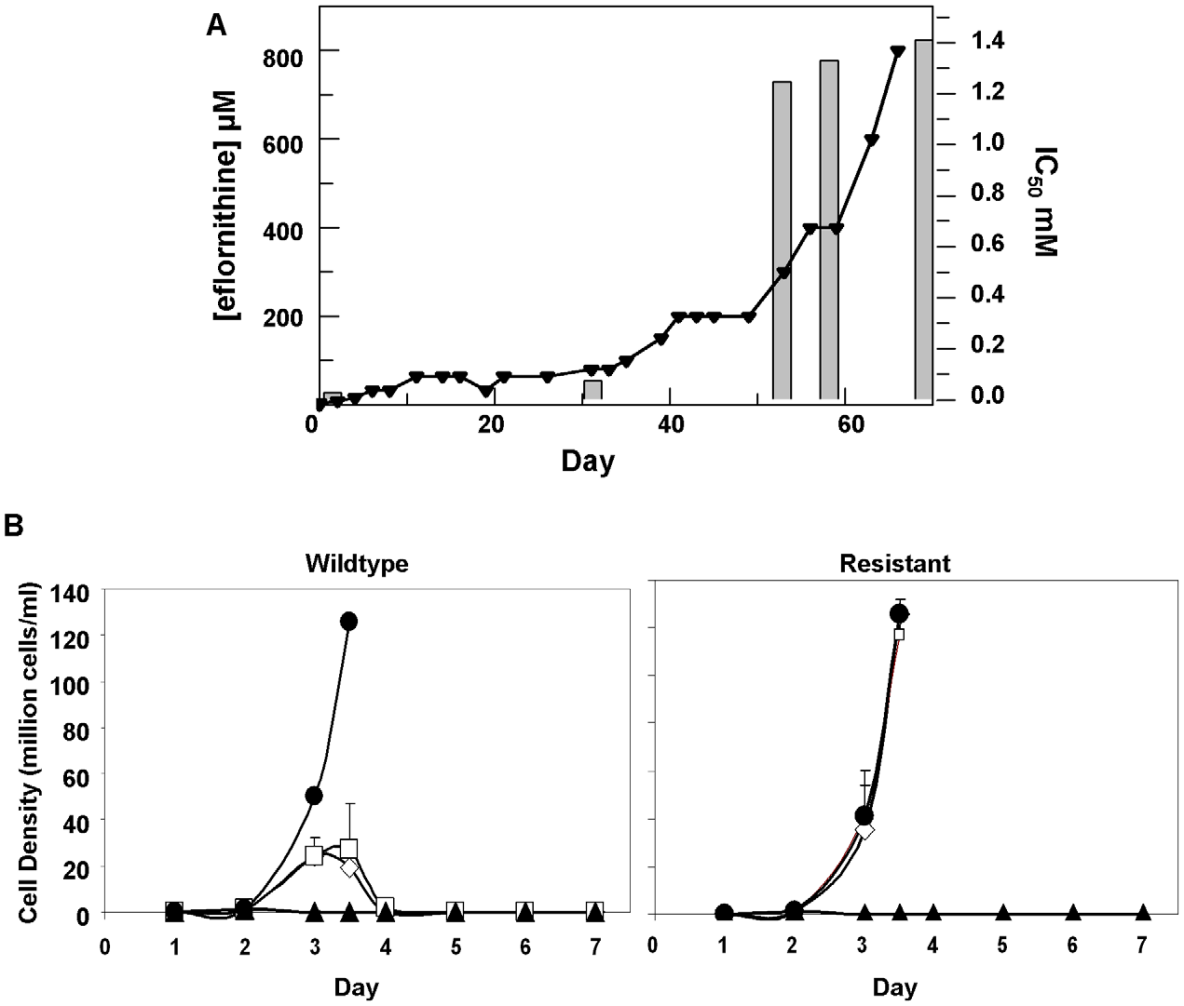
Resistance in *T. brucei brucei (A)*, Selection of eflornithine resistance in *Trypanosoma brucei*. Black triangles and left hand y-axis show the eflornithine concentration in which the parasites grew. Bars and the right hand y-axis show molar IC_50_ values at various stages of the selection process. One clone out of two is shown. *(B)*, Treatment of mice infected with wildtype or eflornithine resistant parasites. Closed circles; untreated, open diamonds; 2% eflornithine, open squares; 5% eflornithine, closed triangles; pentamidine (2mg/ml).

**Figure 2:**
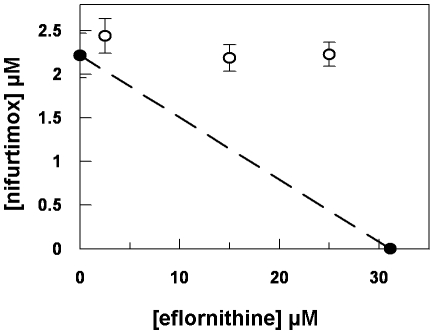
Isobologram analysis of nifurtimox and eflornithine combination. Closed circles show the IC_50_ values of drugs alone. Open circles show the IC_50_ values of the drug combinations.

Eflornithine resistant parasites were derived *in vitro* from a wildtype bloodstream form *T. brucei brucei* strain 427 by growth in increasing concentrations of drug. It took two months (24 passages) to attain a line expressing forty fold less sensitivity to drug, based on the IC_50_ value of eflornithine in the drug sensitive parent strain (Fig. 1*A*) and no growth phenotype was observed. Two independent cell lines were generated in this way. There was no cross-resistance with other currently used trypanocides (Table 1), although there was a significant increase in sensitivity to pentamidine, which we cannot explain at this juncture. The resistant lines also grew in female ICR (Institute for Cancer Research) mice and exhibited resistance to both the minimum curative dose of 2% w/v and a higher 5% w/v eflornithine whilst mice infected with wildtype cells were cured with the lower 2% w/v dose. Resistant cells remained susceptible to pentamidine (4 mg kg^−1^, four daily doses) (Fig. 1*B*). This demonstrates that the *in vitro* selected mechanism for resistance is also operative *in vivo*. Interestingly, isobologram analyses (Fig. 2) revealed that nifurtimox and eflornithine are not synergistic to one another's activity *in vitro*. The average fractional inhibitory concentration (FIC) is used as a measure of interaction between two drugs and is a sum of the IC_50_ of the drug acting in combination divided by the IC_50_ of the drug acting alone. An FIC of 1.5 was recorded for eflornithine and nifurtimox, where a value ≥1.4 is taken as antagonistic [17]). This was a surprise given the theory that eflornithine would deplete cellular trypanothione thus rendering the cells more susceptible to oxidative stress induced by nifurtimox.

**Table 1:** IC_50_ values for known trypanocides on wildtype and eflornithine resistant cell lines.

Trypanocide	Wildtype IC_50_ (nM)	Resistant IC_50_ (nM)	Average R:WT
Suramin (n = 3)	4.6±0.7	4.4±0.4	0.99
Melarsen Oxide (n = 2)	4.3	2.4	0.67
Cymelarsan (n = 2)	6.3	3.7	0.73
Nifurtimox (n = 5)	2,940±600	2,880±300	1.09
Pentamidine (n = 5)	0.43±0.1	0.1±0.04	0.27*
Eflornithine (n = 5)	22,000±3,000	906,000±192,000	41.46*

Number of replicates are in parentheses, numbers represent mean ± s.e.m where appropriate.

*indicates significance at a p = 0.05 level.

### Polyamine pathway metabolite levels are unchanged in eflornithine resistant cells

**Figure 3:**
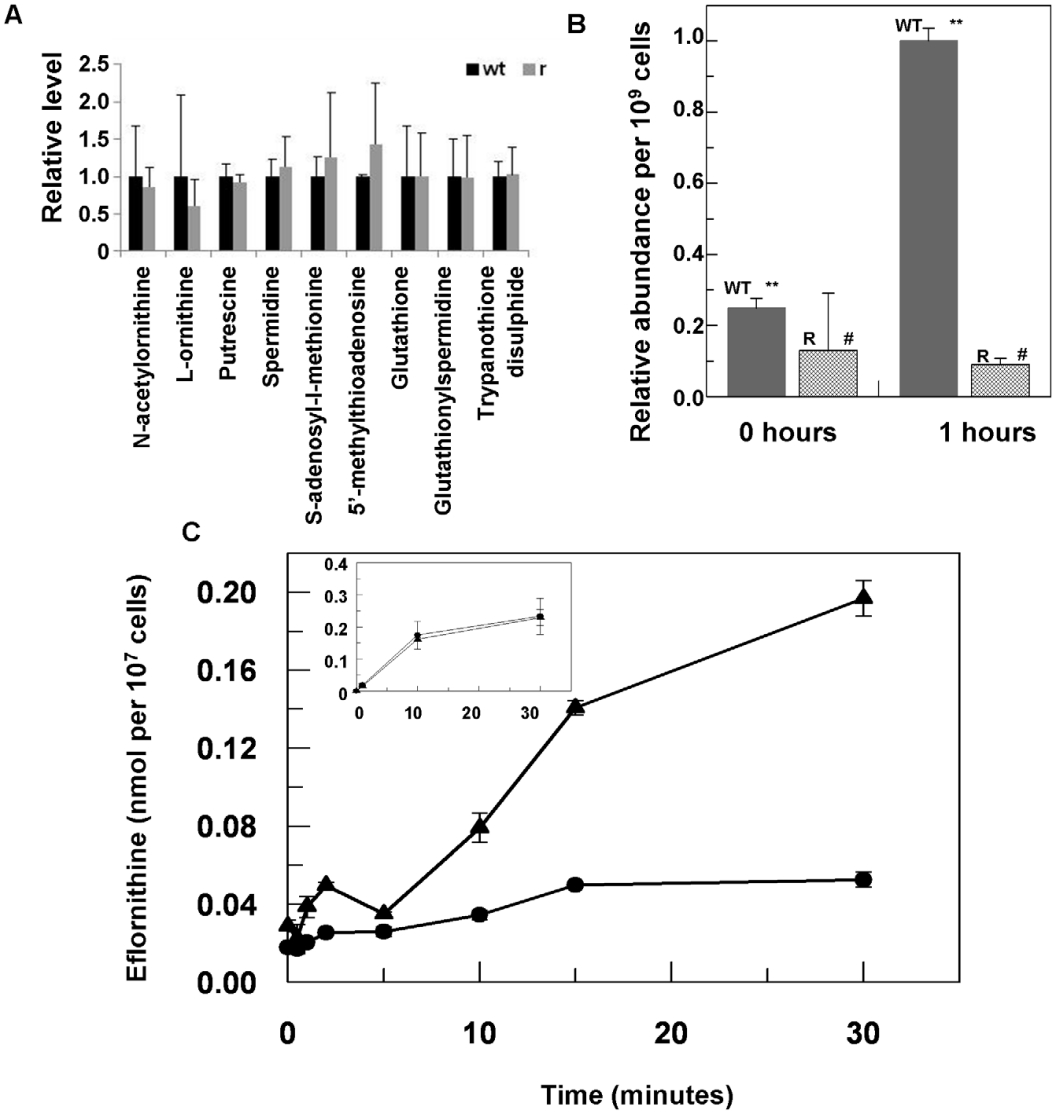
Metabolomic analysis of eflornithine resistance and uptake. *(A)*, Relative abundance of polyamine metabolites in wildtype (WT) and eflornithine resistant (R) cell extracts. *(B)*, Uptake of eflornithine in wildtype (filled bars) and resistant cells (hatched bars) over one hour. Stars indicate a significant difference at a 0.01 level between WT at time 0 and WT after 60 minutes. A hash indicates that R at time 0 and R after 60 minutes show no significant difference at a 0.05 level. *(C)*, Eflornithine uptake in wildtype and resistant cells. ^3^H-eflornithine transported into wildtype (triangles) and resistant (circles) cells was measured over 30 minutes. Measurements are an average of four separate experiments, each with three internal replicates. Error bars are the standard error of the mean. Inset graph shows threonine uptake in the same cell lines. The y-axis shows nmol of threonine per 10^7^ cells. The x-axis shows the time in minutes.

Eflornithine's target is the enzyme ornithine decarboxylase. Alterations to the amino acid composition of proteins is often responsible for drug resistance as variants with diminished ability to bind drug are selected [18]. We therefore amplified the ODC gene from the wildtype and the resistant cell line (DFMOR1 and R2) and found no differences in the sequence or copy number. Earlier work [19] had pointed to possible changes in S-adenosyl methionine and polyamine metabolism relating to refractoriness to eflornithine. We therefore subjected wildtype and resistant cells to untargeted metabolomic analysis to determine whether changes in relative levels of key metabolites could be determined (Figure S1 and Table S1). Significant differences between the untargeted metabolite profiles of wildtype and resistant cells were not apparent using multivariate statistical analysis, nor were changes seen in any of the identified polyamine pathway metabolites including S-adenosyl methionine (Fig. 3*A*). However, in a targeted analysis of eflornithine (m/z = 183.0940) accumulation, it was evident that eflornithine levels were greatly reduced in resistant cells compared to wildtype (Fig. 3*B*). This result indicated that exclusion of drug from the resistant line (DFMOR1) rather than changes to metabolism were responsible for loss of sensitivity.

### Loss of eflornithine accumulation into resistant cells

To determine quantitatively the relative transport rates of the drug in wildtype and resistant cells, ^3^H-eflornithine was used to measure accumulation in each cell type. A greater rate of eflornithine uptake was observed in the wildtype cell line compared to the resistant line (DFMOR1), with around five fold more drug taken into wildtype cells after 30 minutes (Fig. 3*C*).

**Figure 4:**
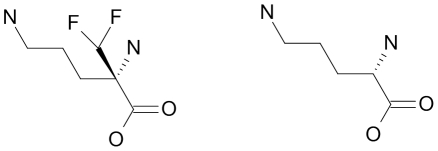
Eflornithine (left) is a derivative of ornithine (right).

**Figure 5:**
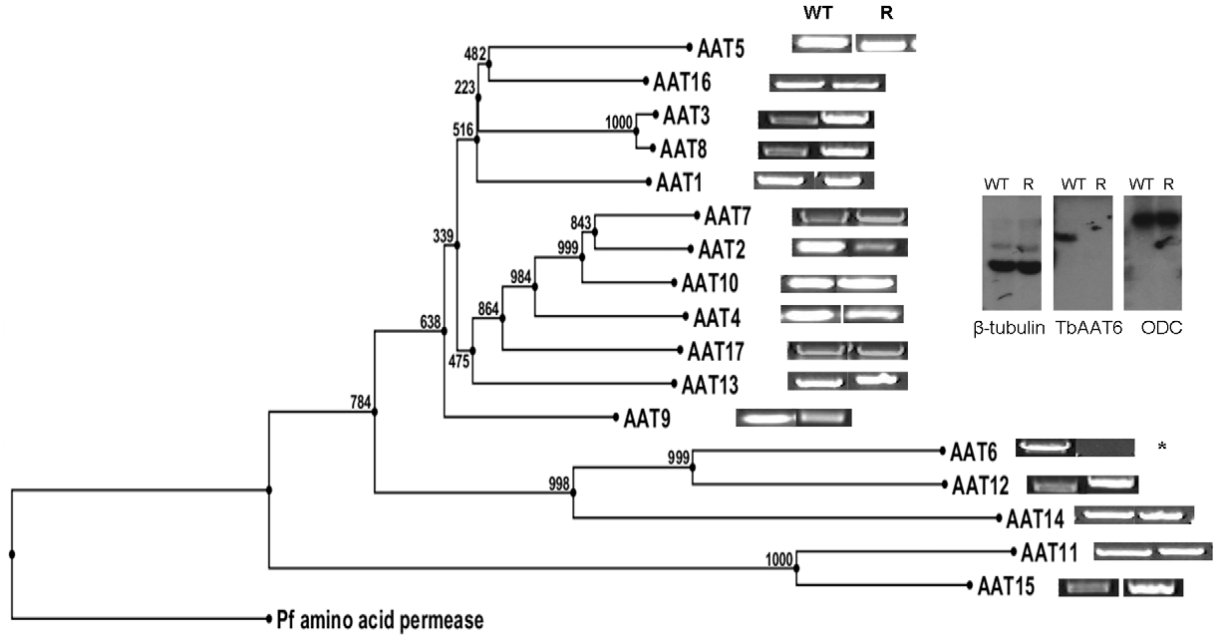
Cladogram of the amino acid transporters predicted to be in *T. brucei* and how amplification of wildtype and resistant cell PCR products of 17 amino acid transporters from the wildtype and resistant cell lines shows *TbAAT6* to be absent. Inset: Southern blotting showed the loss of *TbAAT6* in resistant, but not wildtype cells. *ODC* (ornithine decarboxylase) and *β-tubulin* remained unchanged.

**Figure 6:**
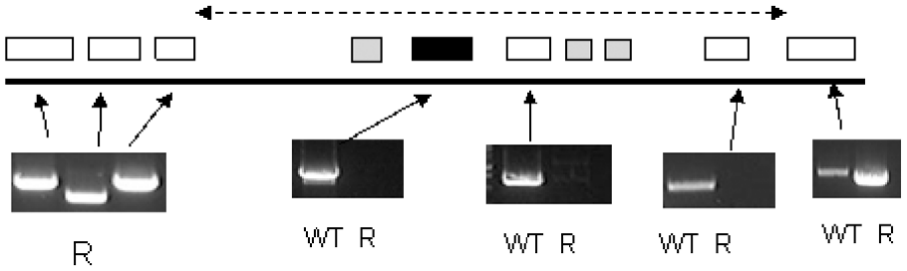
PCR analysis of the region of chromosome 8 housing the single copy *TbAAT6* (black box) in *T. brucei*. An area of DNA is missing including *TbAAT6*. The exact boundary of the missing area is unknown (represented by the dotted line). Genes are (left to right) Tb927.8.5410 (hypothetical), Tb927.8.5420 (hypothetical), Tb927.8.5430 (hypothetical), Tb927.8.5440 (*Tb-24*, a flagellar calcium-binding protein), Tb927.8.5450 (*TbAAT6*), Tb927.8.5460 (*Tb-44* a flagellar calcium-binding protein), Tb927.8.5465 (*Tb-24*, a flagellar calcium-binding protein), Tb927.8.5470 (*Tb-17* a flagellar calcium-binding protein), Tb927.8.5480 (hypothetical), Tb927.8.5490 (hypothetical). Not all of these genes were amplified as *Tb17*, *Tb24* and *Tb44* are repetitive throughout the genome.

These data indicated a transporter phenotype, as seen previously in selection of resistance to melamine based arsenicals [13] and diamidines [14], [16], [20], [21]. As eflornithine is an amino acid analogue (Fig. 4), we hypothesised loss of an amino acid transporter. To test this, members of the amino acid permease gene family (Fig. 5) in the *T. brucei* genome [22] were systematically amplified from both wildtype and each of the two independently selected resistant lines. In each of the independently selected lines only one single copy amino acid transporter gene, *TbAAT6* (Tb927.8.5450), was shown to be absent (Fig. 5). PCR analysis indicated a deletion of this, and surrounding genes, from both resistant lines (DFMOR1, Fig. 6, R2 not shown). This result indicated the possibility that the *TbAAT6* gene could play a role in eflornithine's entry into *T. brucei* and that its loss was responsible for drug resistance. The gene was amplifiable at day 34 (Fig. 1*A*), but by day 50 (Fig. 1*A*) was no longer amplifiable.

### Functional confirmation of a role for TbAAT6 in eflornithine resistance

**Figure 7:**
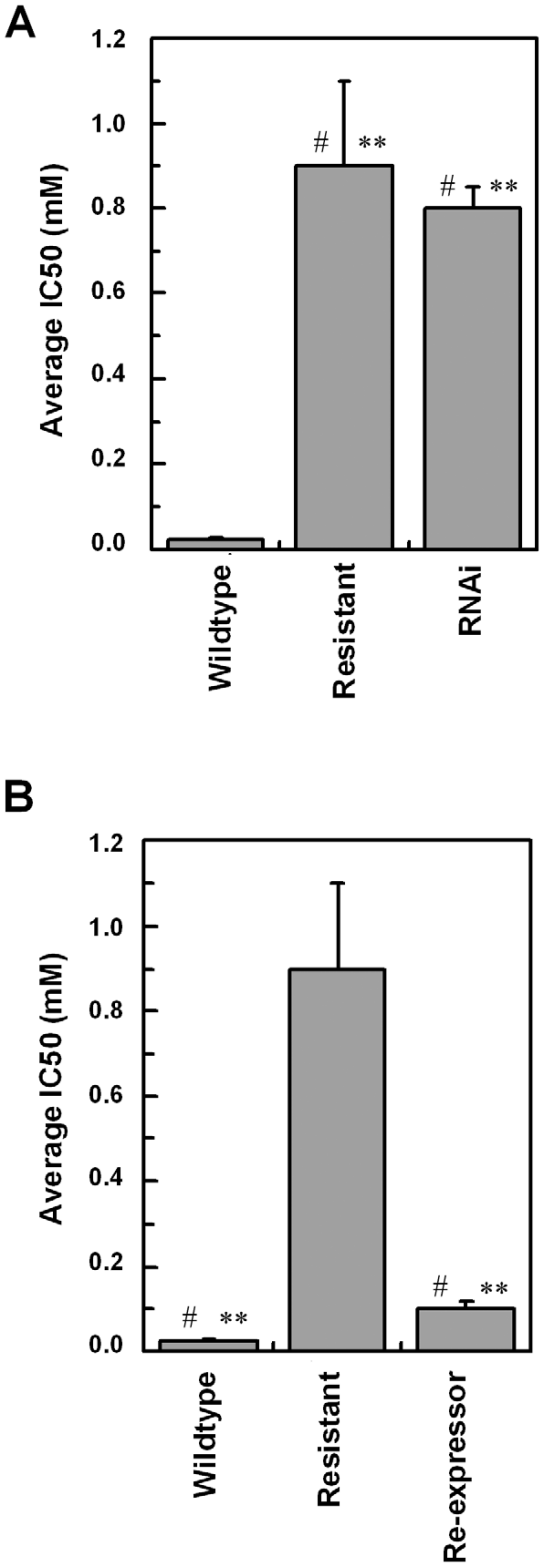
RNAi and re-expression of TbAAT6. *(A)*, RNAi was induced for 12 days and the IC_50_ value to eflornithine measured. Stars indicate significant difference at a 0.05 level compared to wildtype, whereas a hash indicates that RNAi and resistant lines show no significant difference. *(B)*, The IC_50_ value of a constitutive re-expressor of TbAAT6 put into the resistant line. Stars indicate a significant difference at a 0.05 level compared to resistant, whereas a hash indicates that wildtype and re-expressor show no significant difference. Wildtype 24.6±5.8 µM, Resistant 886±200 µM, Re-expressor 111±18 µM, RNAi 773±53 µM. IC_50_ measurements were at least n = 5.

To confirm a role for TbAAT6 in eflornithine resistance we used RNA interference [23] to ablate its expression in *Trypanosoma brucei*. A cloned line was selected and this *TbAAT6* ^RNAi^ mutant became resistant to eflornithine to an extent similar to the lines selected for resistance to the drug (40.1×resistance factor) (Fig. 7*A*) when expression was ablated by addition of tetracycline. Next, we expressed the *TbAAT6* gene in the eflornithine selected trypanosomes using vector pHD676 [24]. Cloned cells in which the gene was re-expressed regained levels of eflornithine sensitivity similar to wildtype (Fig. 7*B*). Loss of expression of TbAAT6 is therefore both necessary and sufficient to confer resistance to eflornithine and its re-expression in defective lines capable of restoring sensitivity, regardless of other changes to the cell.

## Discussion

Human African trypanosomiasis, also known as sleeping sickness in its second stage when parasites have invaded the brain, is a neglected tropical disease [1]. Major epidemics at the end of the twentieth century were brought under control largely through increased efforts in distribution and treatment with the few drugs available to treat the disease [2]. An alarming increase in the incidence of treatment failure with melarsoprol has led to its being replaced with eflornithine as first line treatment for stage 2 HAT [2]. Combination therapy using eflornithine with the nitrofuran, nifurtimox, licensed for use in Chagas' disease has been added to the World Health Organisation's list of essential medicines as part of the nifurtimox-eflornithine combination therapy for HAT [3]. Although several initiatives are underway to develop new drugs for human African trypanosomiasis, none are currently in human trials and a minimum of five years will elapse before a new drug could complete trials and reach the market place. The loss of eflornithine, alone or in the nifurtimox combination, would represent a calamity in terms of sustaining control of HAT.

The data presented here show that resistance to eflornithine is easily selected in the laboratory. Selection of resistance in two independently derived lines led to deletion of the *TbAAT6* gene. Eflornithine uptake was lost indicating that this gene encodes a transporter capable of carrying the drug into trypanosomes. The loss of *TbAAT6* either by gene deletion as observed in the selected drug resistance lines, or by RNAi is sufficient to render trypanosomes over 40 fold less sensitive to eflornithine than wildtype cells. Furthermore, ectopic expression of *TbAAT6* in trypanosomes that have deleted the gene is sufficient to restore wildtype levels of eflornithine sensitivity confirming that loss of *TbAAT6* alone is necessary and sufficient to generate resistance.

We have, as yet, been unable to assign a physiological function to TbAAT6 in African trypanosomes, and this is a topic of ongoing research. However, it is one of a large family of related genes described in the kinetoplastida belong to the amino acid transporter 1 superfamily. Only a few other members of the family have been functionally characterised. These include an arginine transporter in *Leishmania donovani* [25], an arginine transporter in *T. cruzi* [26] and polyamine transporters in *L. major* [27] and *T. cruzi* [28]. The AAT6 gene is not syntenic with genes in *Leishmania spp.* or *T. cruzi*. Furthermore, the evolution of the AAT family [22] makes it impossible, currently, to define specific functionality to any of these transporters based on homology alone.

Previous work with bloodstream and procyclic form trypanosomes also revealed a relative simplicity in selecting eflornithine resistance [11], [12], [29]. In procyclic forms reduced rates of eflornithine uptake were identified [11], [12] with possible changes to other transporters for ornithine and putrescine also suggested. In bloodstream forms reduction in eflornithine uptake was noted in two of six eflornithine refractory *T. b. rhodesiense* lines [29], but in the majority of cases no difference in eflornithine uptake was noted leading the authors to dismiss altered drug uptake as an underlying mechanism for the natural refractoriness of many strains of *T. b. rhodesiense* in the field [30]. Possible changes to S-adenosylmethionine metabolism instead were inferred as being significant in that study [29]. Our metabolomics experiments showed that none of the measured polyamine pathway metabolites differed significantly between wildtype and resistant lines in our study. Furthermore, as noted above, the reduced uptake of eflornithine by trypanosomes lacking TbAAT6, without further requirement of changes in metabolism, is both necessary and sufficient to yield a resistance phenotype without any requirement for changes to metabolic pathways which will be essentially unchanged as drug no longer accumulates to inhibitory levels in trypanosomes. Recently, two groups have employed high throughput RNAi screening to determine whether knockdown of any genes correlate with to resistance to various trypanocides including eflornithine. In both instances, TbAAT6 was implicated in loss of sensitivity to eflornithine (David Horn, London School of Hygiene and Tropical Medicine, personal communication) and Isabel Roditi [31].

Since eflornithine has only recently been implemented as first line treatment for stage two HAT, formal published reports of clinical resistance have not yet appeared, although unpublished data (Enock Matovu (Makerere University), personal communication) points to a substantial increase in eflornithine treatment failures in Northern Uganda. Furthermore, given that the actions of nifurtimox and eflornithine are not synergistic, trypanosomes already bearing resistance, through loss of transport, to eflornithine would effectively be subject to nifurtimox monotherapy even in combination chemotherapy. Nifurtimox resistance has been selected *in vitro* and has been shown to be cross resistant with another emerging trypanocide, fexinidazole, currently in clinical trials [32]. Given nifurtimox's lack of efficiency [33], eflornithine resistance alone is likely to lead to large numbers of treatment failures from the combination. If the loss of *TbAAT6* is involved in resistance in the field, then it will be possible to implement a simple PCR-based test for resistance, allowing for more suitable treatments to be administered.

## Materials and Methods

### Ethics statement

This study was undertaken in adherence to experimental guidelines and procedures approved by the UK Home Office under Project Licence No. 60/3760 as complying with the Animals (Scientific Procedures) Act 2006 entitled Biochemistry, genetics and immunology of parasitic protozoa.

### Culturing bloodstream form trypanosomes

Wildtype 427 bloodstream form trypanosomes were cultured in HMI-9 (Biosera) [34] supplemented with 10% foetal calf serum (Biosera) at 37°C, 5% CO_2_. Eflornithine resistant parasites were selected in increasing concentrations of drug starting at 15 µM. When cells were growing at a rate comparable to wildtype they were cloned by limiting dilution and subcultured into double the drug concentration.

### *In vitro* drug treatment

The Alamar blue assay developed by Raz *et al.* [35] for bloodstream form trypanosomes was used. Bloodstream form parasites were seeded 4×10^4^ cells per ml into a serial dilution of eflornithine (a gift from Pere Simarro, WHO) starting at 20 mM. Plates were incubated for 48 hours at 37°C, 5% CO_2_ then 20 µL Resazurin dye (Sigma) at 0.49 µM was added to each well. Plates were incubated for a further 24 hours then read on a fluorimeter (emission 530, excitation 595) (FLUOstar Optima, BMG Labtech). IC_50_ values were calculated using Graphpad Prism5 Software and defined as the concentration of drug required to diminish fluorescence output by 50%. Significance was determined using an unpaired t-test with a Dunnett's post hoc test. For the isobologram analysis Alamar blue assays were conducted using nifurtimox in serial dilution under eflornithine concentrations of 2.5 µM, 15 µM and 25 µM.

### *In vivo* drug treatment

Four groups of mice (three mice per group) were inoculated with *T. brucei* 427 wildtype and another four groups with one of the selected eflornithine resistant lines (termed DFMOR2). Each inoculum consisted of 1×10^6^ parasites per animal (i.e. 200 µL of 5×10^6^ cells mL^−1^) which was administered via intraperitoneal injection. The groups of mice infected with *T. brucei* 427 wildtype and *T. brucei* eflornithine resistant clones were treated in parallel to each other 24 hours post-infection with the different treatment groups as described below following earlier protocols [30]. (a) Eflornithine 2% w/v for six days in drinking water with the eflornithine solution being refreshed every three days; (b) Eflornithine, 5% w/v for six days in drinking water, with the eflornithine solution being refreshed every three days; (c) Pentamidine 4 mg kg^−1^ injected daily via intraperitoneal route for four days (200 µL per injection); and (d) Untreated (i.e. no treatment administered). The exact dosing of eflornithine was determined by daily water consumption measurements. Parasitaemia levels of each animal were monitored daily via venepunctures and microscopic observations of subsequent blood smears. In instances where infection reaches ∼10^8^ cells mL^−1^ or at the end of the experiment, mice were euthanised using a Schedule 1 method.

### PCR analysis

Genomic DNA was denatured at 94°C for two minutes, followed by 30 cycles of 94°C for 15 seconds, annealing (50–55°C depending on specific oligonucleotide) for 15 seconds and extension at 72°C for 30 seconds/500 bases. A final elongation of 7 minutes was used. See Text S1 for primer sequences used.

### Transfection of trypanosomes

2T1 bloodstream form cells were used to create the RNAi cell line with the pRPaSLi stem loop construct [23]. Eflornithine resistant cells (derived from wildtype 427) were used with the pHD676 [24] construct to create the re-expressor line. Linearised plasmid was transfected into the cells using programme X-001 on an Amaxa Nucleofector II. For the RNAi construct, selection was with hygromycin (15 µg/ml) (Sigma). Cells positive for the re-expression construct were selected with hygromycin (15 µg/ml) (Roche) were added after 24 hours and clones were obtained.

### Amino acid uptake

Uptake was analysed using tritiated substrate and eflornithine accumulation using a mass spectrometry approach. In the mass spectrometry approach cells were harvested in mid-log growth phase and resuspended at 1×10^9^ in HMI-9 with added eflornithine at 0.1 mM. These were incubated for 30 minutes, washed in HMI-9 and quenched in hot ethanol. The cell lysate was then run on the Orbitrap mass spectrometer as detailed below.

Tritiated eflornithine was obtained from Moravek Biochemicals with a specific activity of 1.6 Ci/mmol, 1mCi/ml. Mid-logarithmic growth phase cells were grown up to attain sufficient cell densities to permit use of 2×10^7^ cells per reaction. Cells were washed in CBSS buffer (25 mM HEPES, 120 mM NaCl, 5.4 mM KCl, 0.55 mM CaCl.2H_2_O, 0.5 mM MgSO_4_.7H_2_O, 5.6 mM Na_2_HPO_4_, 11.1 mM D-glucose) and resuspended to a density of 1×10^8^/ml. A rapid oil/stop spin protocol, previously described by Carter & Fairlamb [13], was used. 100 µl of oil (1-Bromodo-decane, density: 1.066 gcm-3) (Aldrich) and 100 µl radiolabelled eflornithine in CBSS buffer was added to 0.5 ml Eppendorf tubes. These were centrifuged briefly to remove bubbles. Cells were added to the tubes at room temperature and centrifuged through the oil at 16, 000 RCF for one minute to stop the uptake after various time points. The resulting cell pellet was flash frozen in liquid nitrogen and the base of the tube containing the pellet was cut into 200 µl of 2% SDS in scintillation vials and left for 30 minutes. Three ml of scintillation fluid was added to each vial and these were left overnight at room temperature. Samples were read on a 1450 microbeta liquid scintillation counter (Perkin Elmer).

### Southern blot

Southern blots performed according to standard procedures [36]. DNA was digested with Eco RI (Promega), blotted using a hybond-N membrane (Amersham) and probed with Easytides ^32^P-ATP (Perkin Elmer) incorporated into *TbAAT6* using the Stratagene Prime-it kit.

### RNA interference

2Ti bloodstream form cells were used to create the RNAi cell line with the pRPaSLi construct [23]. Cells were induced with 1 µg/ml tetracycline for 8 days before calculation of the IC_50_ value.

### Metabolite extraction and analysis

Cultures were kept in log phase growth (below 1×10^6^/ml). Metabolites were extracted from cell cultures simultaneously by two methods.

In method A, cells were centrifuged at 1,250 RCF for 10 minutes and re-suspended in HEPES-free HMI-9 to a density of 1×10^9^ cells/ml. These cells were left to recover in an incubator for 30 minutes before quenching by addition of 80°C ethanol to the cell suspension at a 4∶1 ratio ethanol∶cell suspension. These were left at 80°C for two minutes to allow the cells to lyse and denature any proteins. Extracts were then transferred to ice and left for 5 minutes and vortexed briefly.

In method B, 4×10^7^ cells were rapidly cooled to 4°C by submersion of the flask in a dry ice/ethanol bath, and kept at 4°C for all subsequent steps. The cold cell culture was centrifuged at 1,000 RCF for 10 minutes, supernatant removed, and the pellet washed in 30 mL HEPES-free HMI-9. The washed cells were then centrifuged and the supernatant completely removed. Cell lysis and protein denaturation was achieved by addition of 200 µL of cold chloroform/methanol/water (ratio 1∶3∶1), followed by vigorous mixing for 1 hour at 4°C.

For both methods, extract mixtures were centrifuged for two minutes at 16,000 RCF, 4°C. The supernatant was collected, frozen and stored at −80°C until further analysis.

Samples were analysed on an LTQ Orbitrap mass spectrometer (Thermo Fisher) in positive mode, coupled to HPLC separation using a ZIC-HILIC column (Sequant) according to the method published by Kamleh *et al.* [37]. Each sample was also analysed on an Exactive orbitrap mass spectrometer (Thermo Fisher) in both positive and negative modes (rapid switching), coupled to HPLC with a ZIC-HILIC column. Exactive data was acquired at 25,000 resolution, with spray voltages +4.5kV and −2.6kV, capillary temperature 275°C, sheath gas 20, aux gas 15 and sweep gas 1 unit. Minor adjustments were made to the published HPLC mobile phase gradient as follows: Solvent A is 0.1% formic acid in water, and solvent B is 0.1% formic acid in acetonitrile, 80% B (0 min), 50% B (12 min), 50% B (26 min), 20% B (28 min), 20% B (36 min), 80% B (37 min), 80% B (47 min).

Metabolite identification and relative quantitation was undertaken using ToxID software (Thermo Fisher), by searching for peaks that correspond to the accurate mass of metabolite ions within a 3 ppm window (or 5 ppm window for Exactive data). The metabolite lists were obtained from trypanosome-specific databases in Trypanocyc (metacyc.org) and KEGG (www.genome.jp/kegg/), lipids were excluded from the data analysis. Metabolite levels are expressed as mean peak height from 3 biological replicates. Multivariate statistical analysis comprised a principal component analysis based on putatively identified metabolites, and significance for individual metabolites was calculated by t-test (α = 0.05).

### Cladogram construction

Cladograms were constructed using the CLC genomics workbench software alignment and tree building tools. A neighbour joining algorithm was used and the tree was bootstrapped 1000 times.

## Supporting Information

Text S1 Oligonucleotides used for amplification of TbAAT genes and vector construction.
(0.08 MB DOC)

Figure S1 The mass of each metabolite is shown on the right hand side. The y-axes show relative intensities for each metabolite on exit from the chromatography column.
(0.47 MB TIF)

Table S1 Retention times on the HILIC column along with ratios and p values are shown for each detected metabolite.
(0.06 MB XLS)
