# Supplementary material for: A Molecular Mechanism for Eflornithine Resistance in African Trypanosomes
Source: PLoS Pathog. 2010 Nov 24;6(11):e1001204. doi: 10.1371/journal.ppat.1001204 (PMC2991269; doi:10.1371/journal.ppat.1001204)
Supplement: Text S1 — Oligonucleotides used for amplification of TbAAT genes and vector construction. (0.08 MB DOC) [file ppat.1001204.s001.doc]

| Primer | Sequence | Gene |
| --- | --- | --- |
| AATP2 forward | ATATGGATCCGTGAGTCTATTTATGGCAACT | Tb927.8.7600 |
| AATP2 reverse | ACATCTCGAGGCATGAGTGCACTACAATGGC | Tb927.8.7600 |
| AATP3 forward | ATATGGATCCATTATAATTACTGTTATGACT | Tb927.4.4830 |
| AATP3 reverse | ACATCTCGAGCTCATACGCCGTAATTTGTGA | Tb927.4.4830 |
| AATP4 forward | ATATGGATCCGAAGGCATGTCGAGGGCGCTG | Tb927.8.7680 |
| AATP4 reverse | ACATCTCGAGGAAGCATACGGCCATGGCGAT | Tb927.8.7680 |
| AATP5 forward | ATATGGATCCTCGTGTCTAAATGGGCTTCCG | Tb927.4.4020/Tb927.8.8290 |
| AATP5 reverse | ACATCTCGAGCTTTGGGATGAAGAGACCCAA | Tb927.8.8290 |
| AATP6 forward | ATATGGATCCACCGCGCGGTGGTGCCCTTCC | Tb927.8.4710 |
| AATP6 reverse | ACATCTCGAGTTCACATGACAAAGATAAGCG | Tb927.8.4710 |
| AATP7 forward | ATATGGATCCGCTAATGAGGGAGAAGGGGAA | Tb927.4.4840/4820/4860 |
| AATP7 reverse | ACATCTCGAGGTGCCGAAGAGGTGAATGCCC | Tb927.4.4840/4820/4860 |
| AATP9 forward | ATATGGATCCTGCTCATTTCCTCCGTAACCG | Tb927.8.8300 |
| AATP9 reverse | ACATCTCGAGGTTTCCGGTGTTGAAAAGGAT | Tb927.8.8300 |
| AATP11 forward | ATATGGATCCTCAGCGATCGGCGTTTCGTTC | Tb927.8.7740/Tb927.4.4730 |
| AATP11 reverse | ACATCTCGAGCCAGCCCACGCTTTGGCGTGT | Tb927.8.7740/Tb927.4.4730 |
| AATP12 forward | ATATGGATCCAAACAGATCAATTCCCTGCGC | Tb10.70.0300 |
| AATP12 reverse | ACATCTCGAGACATAATTTGGCAACGAGCCC | Tb10.70.0300 |
| AATP13 forward | ATATGGATCCACATTCTTTACGAAGGTGAGT | Tb927.4.3990 |
| AATP13 reverse | ACATCTCGAGGATCACAATGACAAAATACAC | Tb927.4.3990 |
| AATP14 forward | ATATGGATCCTTTTCCCTGCATATCCTGTCA | Tb11.01.7590 |
| AATP14 reverse | ACATCTCGAGGAACCTGGCACAGCTGCGCTT | Tb11.01.7590 |
| AATP16 forward | ATATGGATCCAAATTTATTTTCGGGCCACCA | Tb927.8.5450 |
| AATP16 reverse | ACATCTCGAGGTCTTCTGATTGCATCCGGTG | Tb927.8.5450 |
| AATP17 forward | ATATGGATCCTGCATGCATTAGTGGTGGTTA | Tb09.211.1760 |
| AATP17 reverse | ACATCTCGAGCCTCCAGGGATCTGGATGAAG | Tb09.211.1760 |
| AATP18 forward | ATATGGATCCGTAAACGTCGGGCTGTGATTG | Tb10.6k15.0450 |
| AATP18 reverse | ACATCTCGAGAATTTCGCACAATGTCACCAC | Tb10.6k15.0450 |
| AATP20 forward | ATATGGATCCGCGATTTCTCACGAGCCTACG | Tb927.4.3930 |
| AATP20 reverse | ACATCTCGAGTACGACACCTCACCACCAAAA | Tb927.4.3930 |
| AATP21 forward | ATATAAGCTTCCTCACTTACTGCGCATATTG | Tb11.01.7500/7520 |
| AATP21 reverse | ACATGGATCCGAGGGTATACTTCAATTAGGT | Tb11.01.7500/7520 |
| AATP22 forward | ATATGGATCCTGATGTGGTAAAGGAAGTGAA | Tb927.8.8220 |
| AATP22 reverse | ACATCTCGAGATAGCCAAGATAATCACCAAC | Tb927.8.8220 |
| Fwd primer for ODC ORF | ATGACCACCAAATCAACCCC | Tb11.01.5300 |
| Rev primer for ODC ORF | TTATGATTTTTGACTTTTCAACTC | Tb11.01.5300 |
| TbAATP16 fragment | GATCGGGCCCGGTACCAAATTTATTTTCGGGCCACC | Tb927.8.5450 |
| TbAATP16 fragment | GATCTCTAGAGGATCCGTCTTCTGATTGCATCCGGT | Tb927.8.5450 |
| TbAATP16 reverse | GCGGCACACCACAGCTCGGA | Tb927.8.5450 |
| TbAATP16 forward | CCAATCGCGTGTTGATACGT | Tb927.8.5450 |
| Tb-44_reverse | GCAGAACCCATCAGTAATGC | Tb927.8.5460 |
| Tb-44_forwards | CCTATGCTATGTTCACGCTG | Tb927.8.5460 |
| Tb927.8.5410_F | TGGACAGCTGAGGCACATAG | Tb927.8.5410 |
| Tb927.8.5410_R | ACGCCTTAGTTCCTTGAGCA | Tb927.8.5410 |
| Tb927.8.5420_F | TCCTCGGTATAAGCCGATTG | Tb927.8.5420 |
| Tb927.8.5420_R | TCAACTGTTGGGTTTCCACA | Tb927.8.5420 |
| Tb927.8.5430_F | ATGGGCAACAACGGAAGTAG | Tb927.8.5430 |
| Tb927.8.5430_R | GTTGTGATACCGGGACAACC | Tb927.8.5430 |
| Tb927.8.5480_F | CAGCAACTGAGATGAAGGCA | Tb927.8.5480 |
| Tb927.8.5480_R | CGCGTCAAACTTCTTGAACA | Tb927.8.5480 |
| Tb927.8.5490_R | AAAACGAGAGCCAACTCGAA | Tb927.8.5490 |
| Tb927.8.5490_R | GTCAGCAAGCGCAGTGATTA | Tb927.8.5490 |
