# Supplementary figures and images for: A Molecular Mechanism for Eflornithine Resistance in African Trypanosomes
Source: PLoS Pathog. 2010 Nov 24;6(11):e1001204. doi: 10.1371/journal.ppat.1001204 (PMC2991269; doi:10.1371/journal.ppat.1001204)

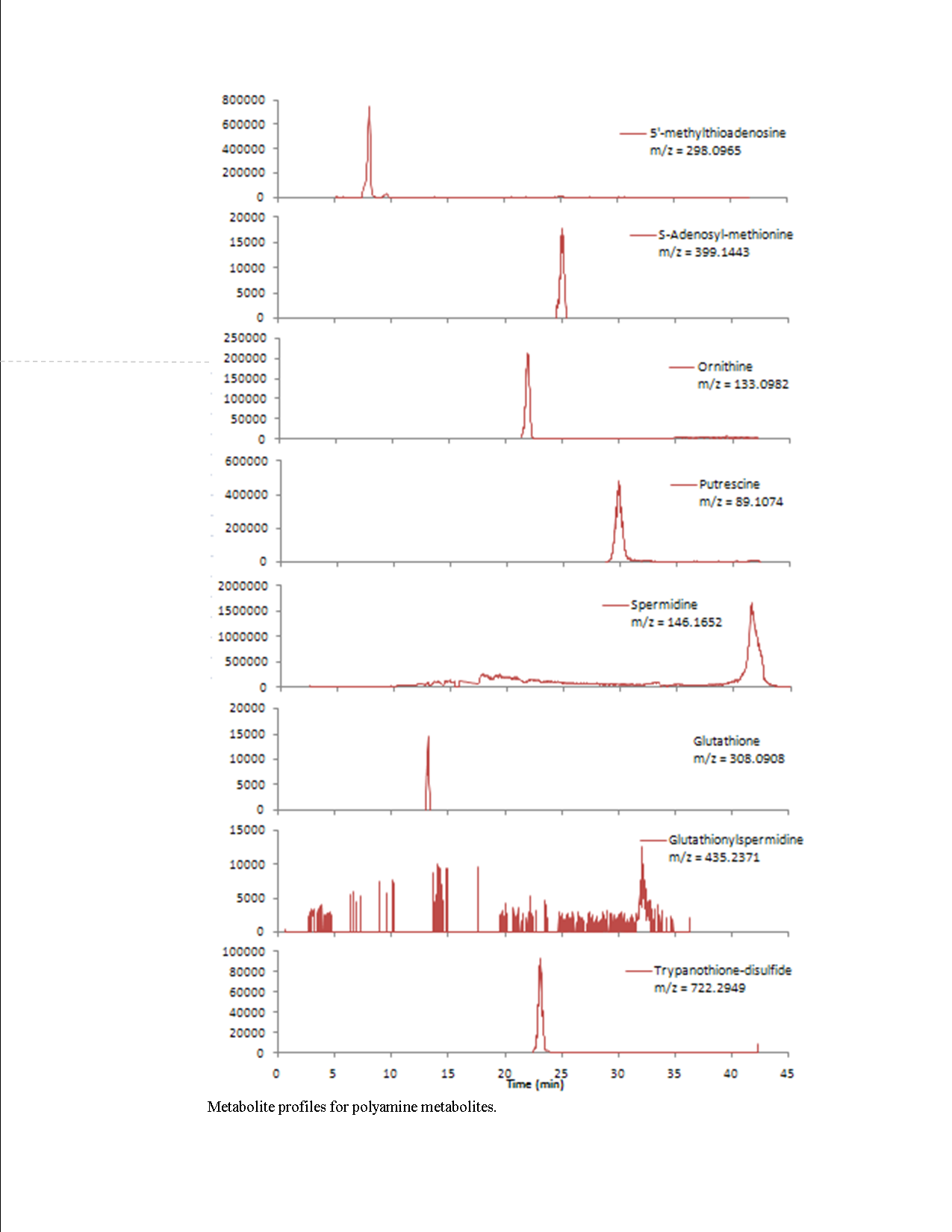

Supplement: Figure S1 — The mass of each metabolite is shown on the right hand side. The y-axes show relative intensities for each metabolite on exit from the chromatography column. (0.47 MB TIF) [file ppat.1001204.s002.tif]
